# Supplementary material for: Search performance and octopamine neuronal signaling mediate parasitoid induced changes in Drosophila oviposition behavior
Source: Nat Commun. 2022 Aug 2;13:4476. doi: 10.1038/s41467-022-32203-5 (PMC9345866; doi:10.1038/s41467-022-32203-5)
Supplement: Supplementary file 1 — Supplementary Information [file 41467_2022_32203_MOESM1_ESM.pdf]

Supplementary information for

Search performance and octopamine neuronal signaling mediate parasitoid induced changes in *Drosophila* oviposition behavior

Supplementary Figure 1 -- 2
Supplementary Figure 2--- 4
Supplementary Figure 3--- 6
Supplementary Figure 4--- 7
Supplementary Figure 5--- 8
Supplementary Figure 6--- 9
Supplementary Figure 7--- 10
Supplementary Figure 8--- 11
Supplementary Figure 9--- 12
Supplementary Figure 10-- 13
Supplementary Figure 11-- 14
Supplementary Figure 12-- 15
Supplementary Table 1 --- 16

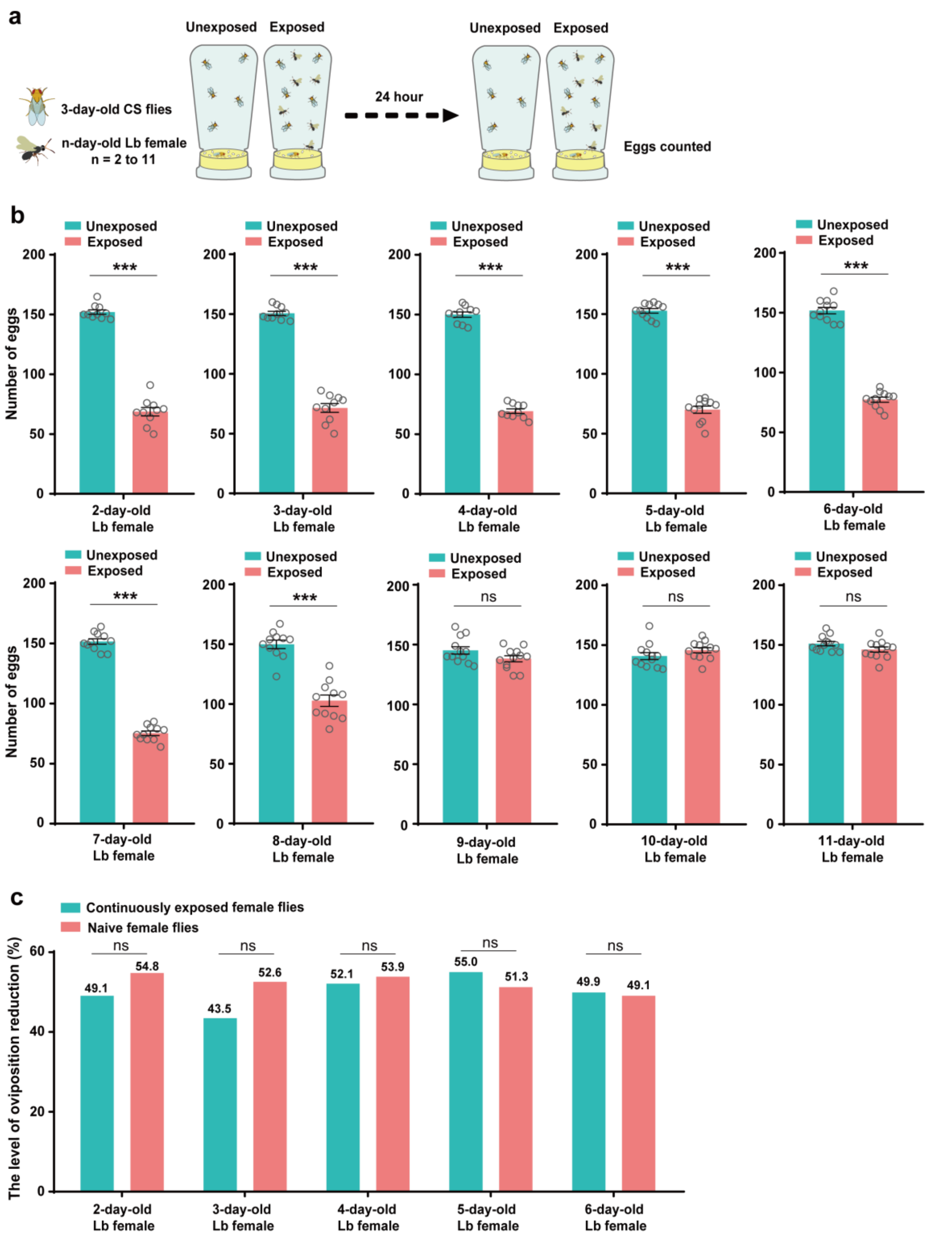

Supplementary Figure 1 The egg laying of naive female flies after exposure to different aged female wasps.

a Oviposition assay design for naive female flies that were independently housing with different aged Lb females for 24 h. Each bottle contained twenty Canton-S (CS) female flies and five CS male flies, either with twenty different aged female Lb wasps (exposed) or with no wasps

(unexposed).Eggs were counted at 24 h. **b** The number of eggs laid by the unexposed and exposed CS flies. The experiment was performed at least ten times. Data represent the mean ± SEM. Significance was determined by two-sided unpaired Student’s t test, p values are indicated in Source Data file (***$p < 0.001$; ns, not significant). **c** The oviposition reduction level of naive flies and continuously exposed flies after housing with the same aged female wasps in the experiments of Fig. 1b and Supplementary Figure 1b. Significance was determined by Fisher nonparametric exact test, p values are indicated in Source Data file (ns, not significant). Source data are provided as a Source Data file.

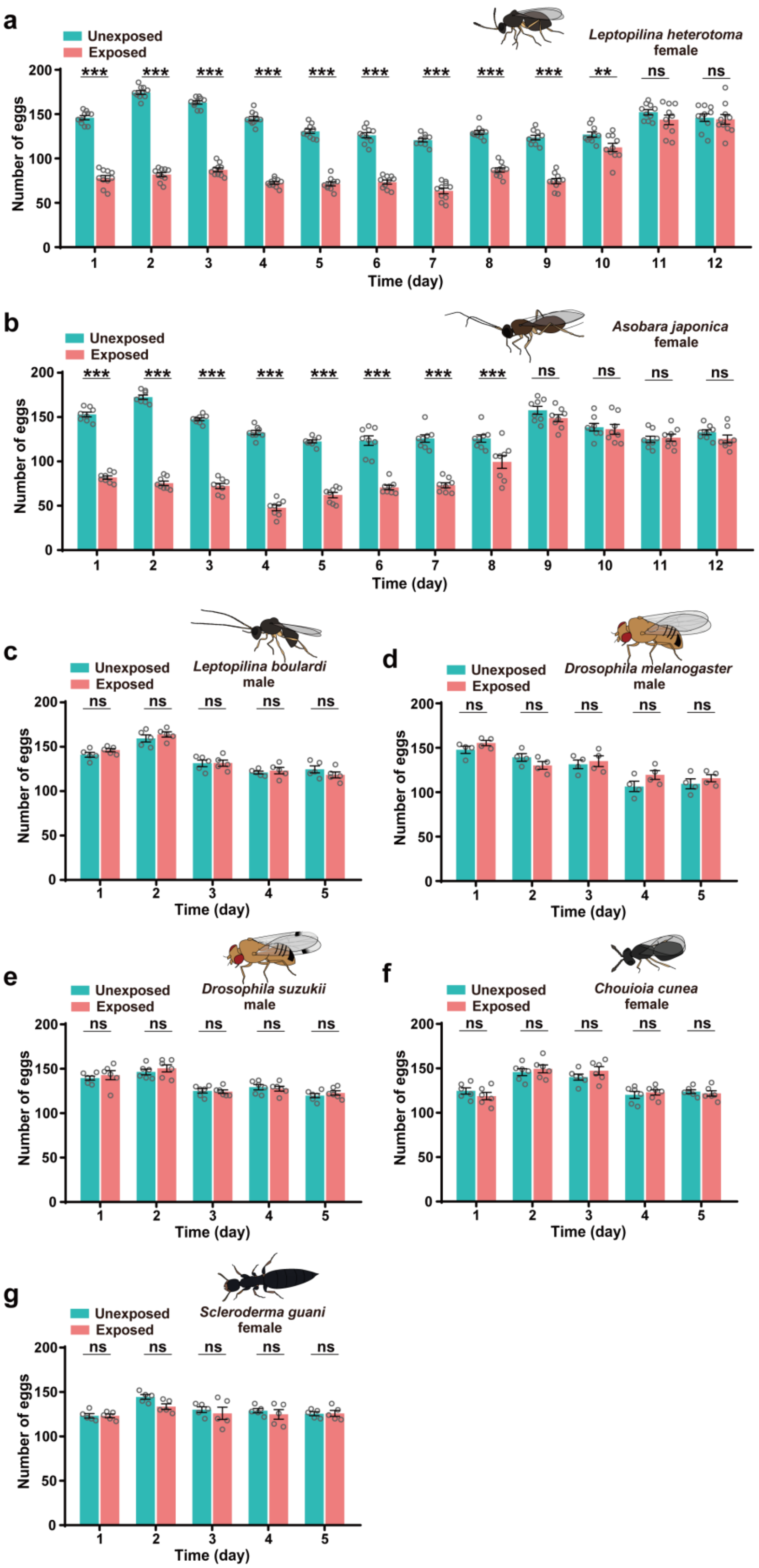

Supplementary Figure 2 Effect of exposure to other insect species on egg laying of *D. melanogaster*.

a The daily number of eggs laid by the unexposed and exposed CS female flies. Flies were exposed to *L. heterotoma* female wasps for 12 days. The experiment was performed ten times. Data represent the mean ± SEM. Significance was determined by two-way ANOVA with Sidak's multiple comparisons test, p values are indicated in Source Data file (**p < 0.01; ***p < 0.001; ns, not significant). **b** The daily number of eggs laid by the unexposed and exposed CS female flies. Flies were exposed to *A. japonica* female wasps for 12 days. The experiment was performed eight times. Data represent the mean ± SEM. Significance was determined by two-way ANOVA with Sidak's multiple comparisons test, p values are indicated in Source Data file (***p < 0.001; ns, not significant). **c** The daily number of eggs laid by the unexposed and exposed CS female flies. Flies were exposed to *L. boulardi* male wasps for 5 days. The experiment was performed five times. Data represent the mean ± SEM. Significance was determined by two-way ANOVA with Sidak's multiple comparisons test, p values are indicated in Source Data file (ns, not significant). **d** The daily number of eggs laid by the unexposed and exposed CS female flies. Flies were exposed to *D. melanogaster* males for 5 days. The experiment was performed four times. Data represent the mean ± SEM. Significance was determined by two-way ANOVA with Sidak's multiple comparisons test, p values are indicated in Source Data file (ns, not significant). **e** The daily number of eggs laid by the unexposed and exposed CS female flies. Flies were exposed to *D. suzukii* males for 5 days. The experiment was performed six times. Data represent the mean ± SEM. Significance was determined by two-way ANOVA with Sidak's multiple comparisons test, p values are indicated in Source Data file (ns, not significant). **f** The daily number of eggs laid by the unexposed and exposed CS female flies. Flies were exposed to *C. cunea* female wasps for 5 days. The experiment was performed six times. Data represent the mean ± SEM. Significance was determined by two-way ANOVA with Sidak's multiple comparisons test, p values are indicated in Source Data file (ns, not significant). **g** The daily number of eggs laid by the unexposed and exposed CS female flies. Flies were exposed to *S. guani* female wasps for 5 days. The experiment was performed five times. Data represent the mean ± SEM. Significance was determined by two-way ANOVA with Sidak's multiple comparisons test, p values are indicated in Source Data file (ns, not significant). Flies aged 3 days post-eclosion and wasps aged 2 days post-emergence were used. Source data are provided as a Source Data file.

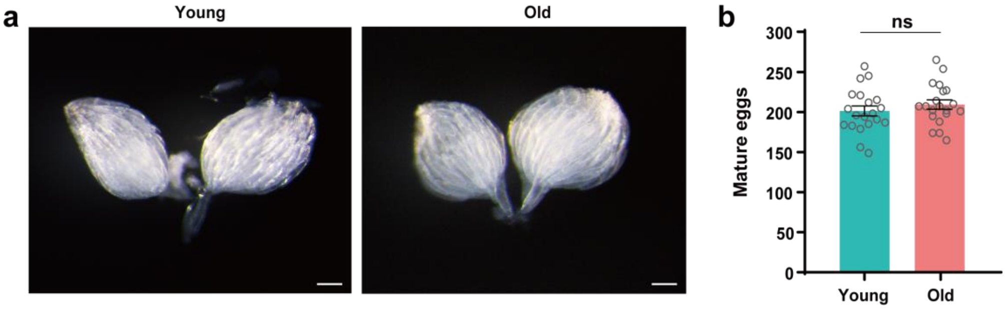

Supplementary Figure 3 The size of ovaries and number of mature eggs in young and old Lb females.

a Images of ovaries from young (4-day-old) and old (12-day-old) Lb female wasps. Three biologically independent experiments were performed. Scale bars: 100 μm. **b** The number of mature eggs per ovary in young (4-day-old) and old (12-day-old) Lb female wasps. n = 20 per group. Data represent the mean ± SEM. Significance was determined by two-sided unpaired Student's t test. (p = 0.3444; ns, not significant). Source data are provided as a Source Data file.

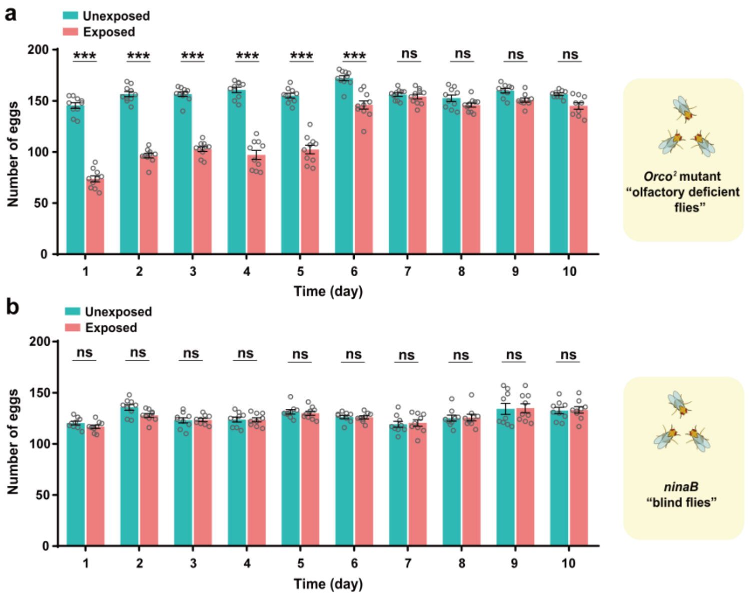

Supplementary Figure 4 Vision is important to the reduced oviposition.

a The daily number of eggs of olfactory-deficient *Orco*^{2} mutant flies exposed to Lb female wasps compared to that of unexposed flies. Flies were exposed to wasps for a testing period of 10 days. The experiment was performed at least nine times. Data represent the mean ± SEM. Significance was determined by two-way ANOVA with Sidak's multiple comparisons test, p values are indicated in Source Data file (***p < 0.001; ns, not significant) **b** The daily number of eggs of blind *nianB* flies exposed to Lb female wasps compared to unexposed flies. Flies were exposed to wasps for a testing period of 10 days. The experiment was performed nine times. Data represent the mean ± SEM. Significance was determined by two-way ANOVA with Sidak's multiple comparisons test, p values are indicated in Source Data file (ns, not significant). Source data are provided as a Source Data file.

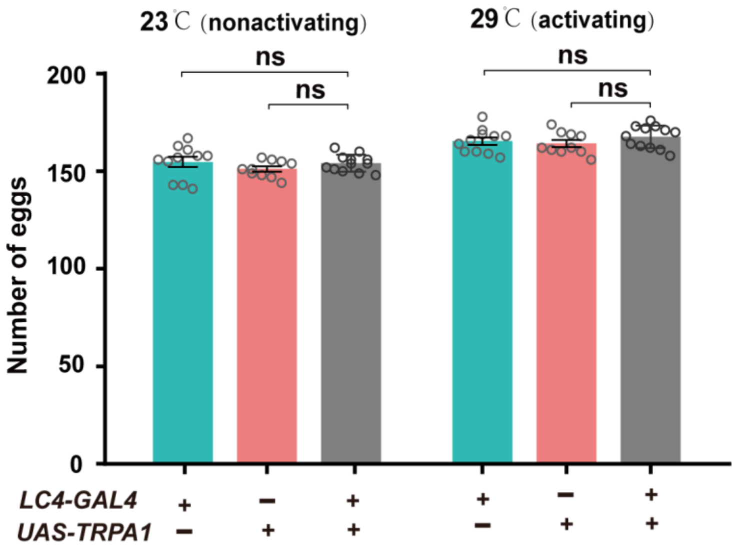

Supplementary Figure 5 Activation of LC4 neurons does not induce the egg reduction. The number of eggs laid by *D. melanogaster* female flies, including *LC42-GAL4*, *UAS-TRPA1*, and *LC4-GAL4>UAS-TRPA1* genotype flies in 24 h. The temperature of 23 °C is nonactivating, and 29 °C increases the activity of LC4 neurons. The experiment was performed at least ten times. Data represent the mean ± SEM. Significance was determined by two-way ANOVA with Sidak's multiple comparisons test, p values are indicated in Source Data file (ns, not significant). Source data are provided as a Source Data file.

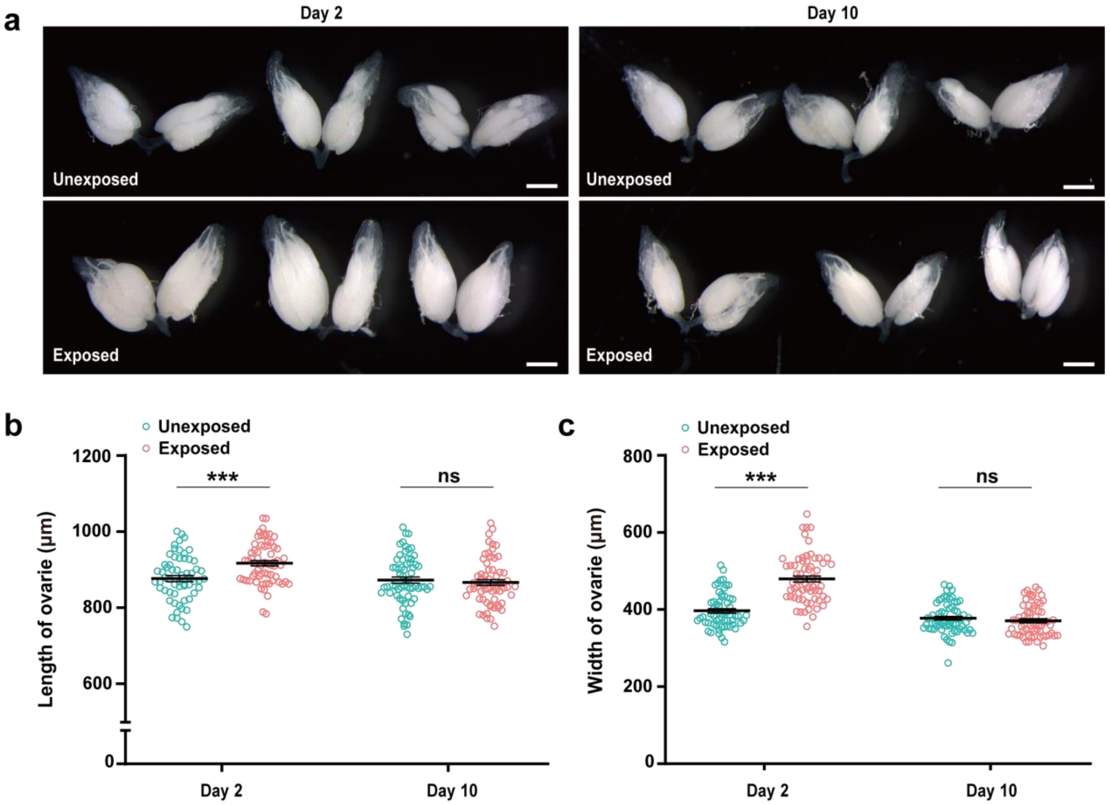

Supplementary Figure 6 Exposure to wasps affects fly ovary size.

a Images of ovaries from the exposed and unexposed flies harvested on Day 2 and Day 10. Three biologically independent experiments were performed. Scale bars, 400 μm. **b** The length of the ovaries from the exposed and unexposed flies harvested on Day 2 and Day 10. Left to right: n = 60, 64, 63 and 63 biologically independent ovaries. Data represent the mean ± SEM. Significance was determined by two-way ANOVA with Sidak's multiple comparisons test, p values are indicated in Source Data file (***p < 0.001; ns, not significant). **c** The width of the ovaries from the exposed and unexposed flies harvested on Day 2 and Day 10. Left to right, n = 60, 64, 63 and 63 biologically independent ovaries. Data represent the mean ± SEM. Significance was determined by two-way ANOVA with Sidak's multiple comparisons test, p values are indicated in Source Data file (***p < 0.001; ns, not significant). Source data are provided as a Source Data file.

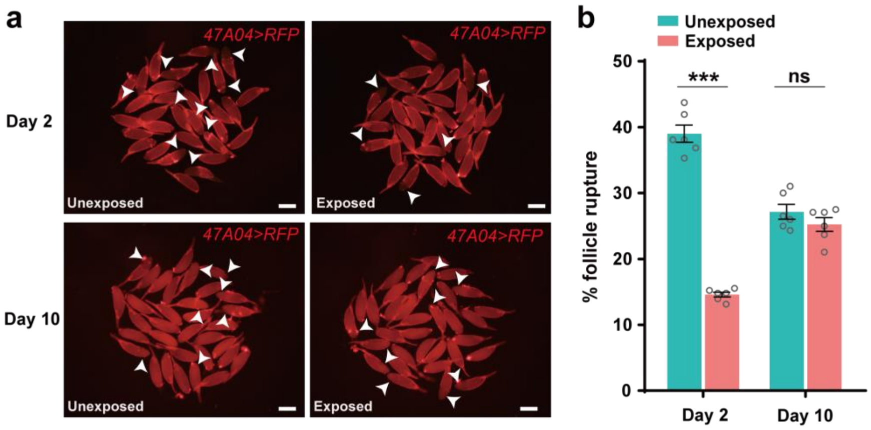

Supplementary Figure 7 Wasp exposure decreases follicle rupture.
a Images of mature oocytes partly (follicle rupture, white arrowhead) or fully covered by follicular cells, which were marked by *47A10-Gal4>UAS-RFP* (*47A10-Gal4* driving *UAS-RFP*). Scale bars: 400 µm. **b** Percent of follicle rupture in the unexposed and exposed flies on Day 2 and Day 10. n = 6 per group; ~30 mature oocytes per replicate. Data represent the mean ± SEM. Significance was determined by two-way ANOVA with Sidak's multiple comparisons test, p values are indicated in Source Data file (***p < 0.001; ns, not significant). Source data are provided as a Source Data file.

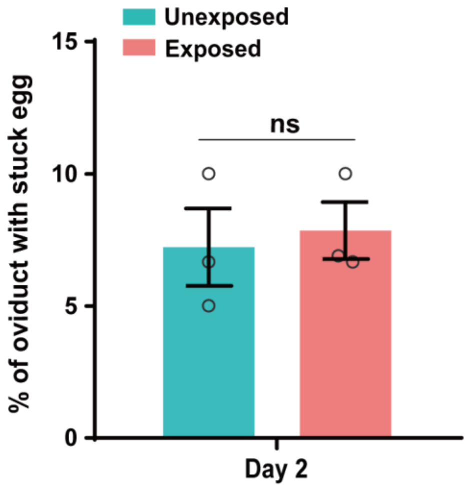

Supplementary Figure 8 Exposure to wasps does not cause eggs to stick in the oviduct. The percent of oviduct in unexposed and exposed female flies with stuck mature eggs on Day 2. Left to right, n = 90 and n = 79, respectively. The experiment was performed three times. Data represent the mean ± SEM. Significance was determined by two-sided unpaired Student's *t* test (p = 0.7459; ns, not significant). Source data are provided as a Source Data file.

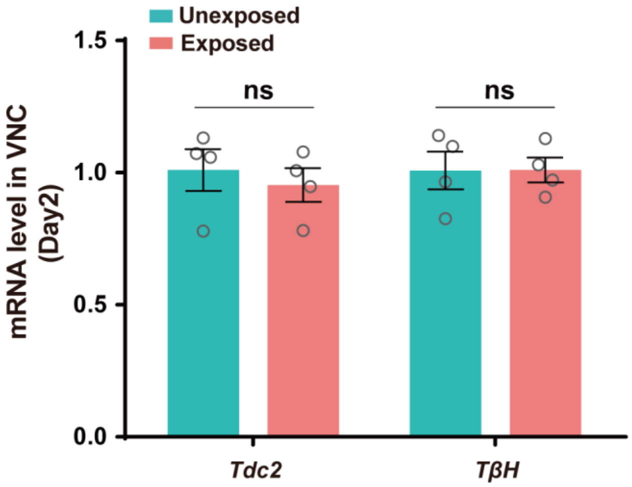

Supplementary Figure 9 Exposure to wasps had no effect on the *Tdc2* and *Tβh* mRNA levels of blind flies.

Quantification of *Tdc2* and *Tβh* mRNA levels in the VNC of the unexposed and exposed *GMR-grim* female flies on Day 2. n = 4 per group. Data represent the mean ± SEM. Significance was determined by two-sided unpaired Student's *t* test, p values are indicated in Source Data file (ns, not significant). Source data are provided as a Source Data file.

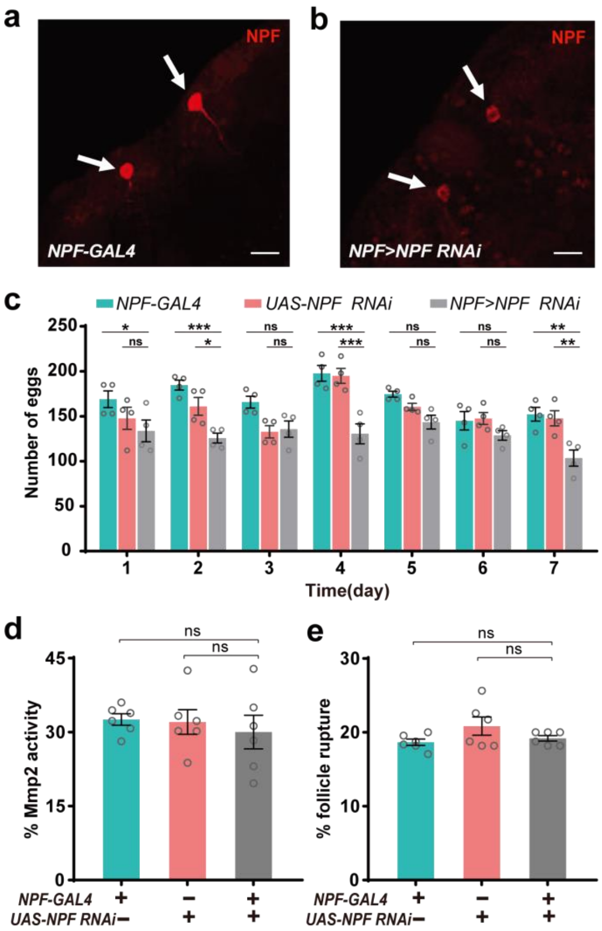

Supplementary Figure 10 OA and NPF neuronal signaling act independently to elicit distinct behavioral responses to the presence of parasitic wasps.

a Representative image of NPF (red) immunolocalization in the *NPF-GAL4* (control) fly brain. Three biologically independent experiments were performed. Scale bars: 20 μm. **b** Representative image of NPF (red) immunolocalization in the *NPF-GAL4>NPF RNAi* fly brain. Three biologically independent experiments were performed. Scale bars, 20 μm. **c** The daily number of eggs laid by *D. melanogaster* female flies, including *NPF-GAL4*, *UAS-NPF RNAi*, and *NPF-GAL4>NPF RNAi* genotypes. The experiment was performed four times. Data represent the mean ± SEM. Significance was determined by two-way ANOVA with Sidak's multiple comparisons test, p values are indicated in Source Data file (*p < 0.05; **p < 0.01; ***p < 0.001; ns, not significant). **d** Percent of mature follicles from ovaries of *NPF-GAL4*, *UAS-NPF RNAi*, and *NPF-GAL4>NPF RNAi* flies with Mmp2 activity. n = 6 per group; ~30 mature oocytes per replicate. Data represent the mean ± SEM. Significance was determined by one-way ANOVA with Sidak's multiple comparisons test, p values are indicated in Source Data file (ns, not significant). **e** Percent of oocytes from *NPF-GAL4*, *UAS-NPF RNAi*, and *NPF-GAL4>NPF RNAi* flies with follicle rupture. n = 6 per group; ~30 mature oocytes per replicate. Data represent the mean ± SEM. Significance was determined by Kruskal-Wallis test with Dunn's multiple comparisons test, p values are indicated in Source Data file (ns, not significant). Source data are provided as a Source Data file.

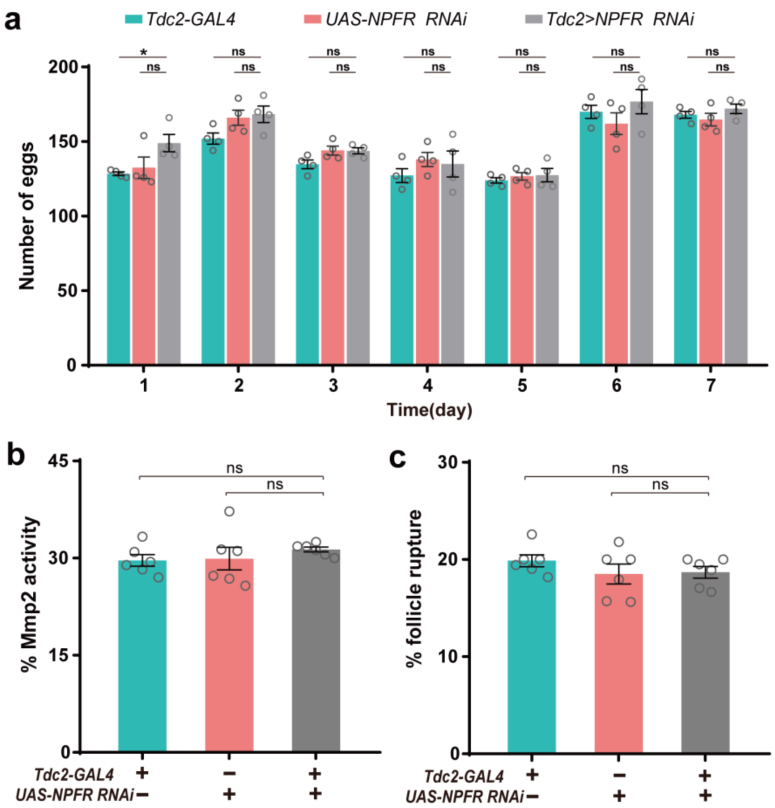

Supplementary Figure 11 The OA-mediated and NPF-mediated signaling pathways are independent.

a The daily number of eggs laid by *D. melanogaster* female flies, including *Tdc2-GAL4*, *UAS-NPFR RNAi*, and *Tdc2>NPFR RNAi* genotypes. The experiment was performed four times. Data represent the mean ± SEM. Significance was determined by two-way ANOVA with Sidak's multiple comparisons test, p values are indicated in Source Data file (*$p < 0.05$; ns, not significant). **b** Percent of mature follicles from ovaries of the *Tdc2-GAL4*, *UAS-NPFR RNAi*, and *Tdc2>NPFR RNAi* flies with Mmp2 activity. n = 6 per group; ~30 mature oocytes per replicate. Data represent the mean ± SEM. Significance was determined by one-way ANOVA with Sidak's multiple comparisons test, p values are indicated in Source Data file (ns, not significant). **c** Percent of mature follicles from ovaries of the *Tdc2-GAL4*, *UAS-NPFR RNAi*, and *Tdc2>NPFR RNAi* flies with follicle rupture. n = 6 per group; ~30 mature oocytes per replicate. Data represent the mean ± SEM. Significance was determined by one-way ANOVA with Sidak's multiple comparisons test, p values are indicated in Source Data file (ns, not significant). Source data are provided as a Source Data file.

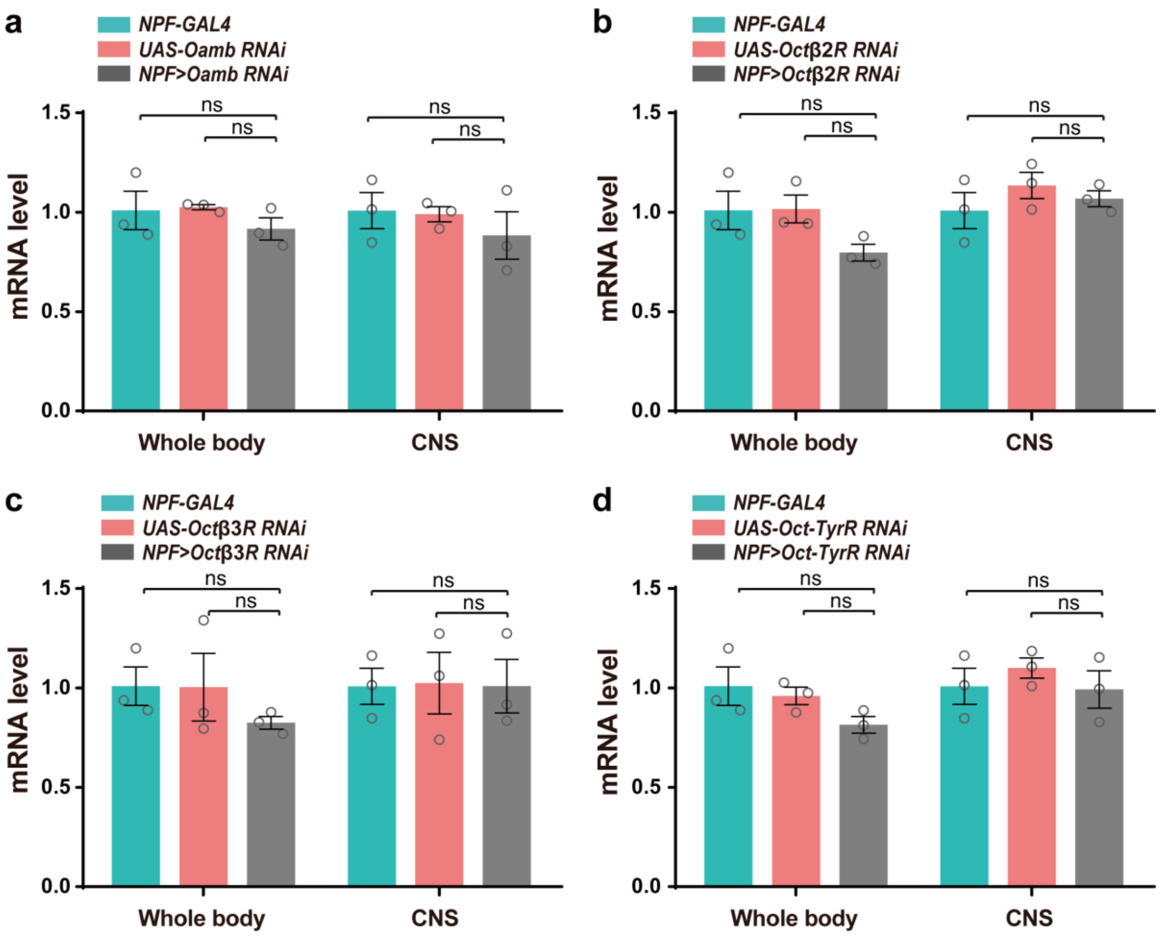

Supplementary Figure 12 OA ablation does not change the NPF levels.

a Quantification of *NPF* mRNA levels in the whole body and central nervous system (CNS) of the *NPF-GAL4>Oamb RNAi* and control female flies. n = 3 per group. Data represent the mean ± SEM. Significance was determined by one-way ANOVA with Sidak's multiple comparisons test, p values are indicated in Source Data file (ns, not significant) **b** Quantification of *NPF* mRNA levels in the whole body and CNS of the *NPF-GAL4>Octβ2R RNAi* and control female flies. n = 3 per group. Data represent the mean ± SEM. Significance was determined by one-way ANOVA with Sidak's multiple comparisons test, p values are indicated in Source Data file (ns, not significant) **c** Quantification of *NPF* mRNA levels in the whole body and CNS of the *NPF-GAL4>Octβ3R RNAi* and control female flies. n = 3 per group. Data represent the mean ± SEM. Significance was determined by one-way ANOVA with Sidak's multiple comparisons test, p values are indicated in Source Data file (ns, not significant) **d** Quantification of *NPF* mRNA levels in the whole body and CNS of the *NPF-GAL4>Oct-TyrR RNAi* and control female flies. n = 3 per group. Data represent the mean ± SEM. Significance was determined by one-way ANOVA with Sidak's multiple comparisons test, p values are indicated in Source Data file (ns, not significant). Source data are provided as a Source Data file.

Supplementary Table S1 The primers used in this study.

Name	Sequence (5’-3’)
GFP RNAi F	TAATACGACTCACTATAGGGCTTCTCGTTGGGGTCTTTGCT
GFP RNAi R	TAATACGACTCACTATAGGGCAGTGCTTCAGCCGCTACCC
Lb *Orco* RNAi F	TAATACGACTCACTATAGGGATGTCGATGATTTAACAGCAAAT
Lb *Orco* RNAi R	TAATACGACTCACTATAGGGAATGTGTTTCATTCCATGACTCG
Tubulin F	GTGGACTCAGTGCTCGATGT
Tubulin R	AATCAGCAGGGTTCCCATAC
Tdc2 F	GTGGACTTTGCCAACGAGTT
Tdc2 R	ATCTTCGGATCGCTGACCAT
Tβh F	ACAATGTACGTGGTTTGGGC
Tβh R	AGTATCTTGTCGGCCCGTAG
NPF F	CTCCGCGAAAGAACGATGTCAACAC
NPF R	CCTCAGGATATCCATCAGCGATCCG
Lb *Tubulin* F	CCAATTGACTCATTCACTTGGC
Lb *Tubulin* R	GTGCTTCGTTGTCAATGCAATA
Lb *Orco* F	CGCAACATGTCTTGCAGTAT
Lb *Orco* R	GCAAACAACAACCACGAACA
