# Supplementary material for: Search performance and octopamine neuronal signaling mediate parasitoid induced changes in Drosophila oviposition behavior
Source: Nat Commun. 2022 Aug 2;13:4476. doi: 10.1038/s41467-022-32203-5 (PMC9345866; doi:10.1038/s41467-022-32203-5)
Supplement: Supplementary file 3 — Description of Additional Supplementary Files [file 41467_2022_32203_MOESM3_ESM.pdf]

Description of Additional Supplementary Files

Supplementary Movie 1: The stereotyped search behavior of Lb female wasps on fly food substrate.
