# Supplementary material for: Search performance and octopamine neuronal signaling mediate parasitoid induced changes in Drosophila oviposition behavior
Source: Nat Commun. 2022 Aug 2;13:4476. doi: 10.1038/s41467-022-32203-5 (PMC9345866; doi:10.1038/s41467-022-32203-5)
Supplement: Supplementary file 5 — Reporting Summary [file 41467_2022_32203_MOESM5_ESM.pdf]

nature portfolio

Corresponding author(s): Jianhua Huang
Last updated by author(s): Jun 30, 2022

Reporting Summary

Nature Portfolio wishes to improve the reproducibility of the work that we publish. This form provides structure for consistency and transparency in reporting. For further information on Nature Portfolio policies, see our Editorial Policies and the Editorial Policy Checklist.

Statistics

For all statistical analyses, confirm that the following items are present in the figure legend, table legend, main text, or Methods section.

n/a	Confirmed	
☐	☒	The exact sample size (*n*) for each experimental group/condition, given as a discrete number and unit of measurement
☐	☒	A statement on whether measurements were taken from distinct samples or whether the same sample was measured repeatedly
☐	☒	The statistical test(s) used AND whether they are one- or two-sided<br>*Only common tests should be described solely by name; describe more complex techniques in the Methods section.*
☒	☐	A description of all covariates tested
☐	☒	A description of any assumptions or corrections, such as tests of normality and adjustment for multiple comparisons
☐	☒	A full description of the statistical parameters including central tendency (e.g. means) or other basic estimates (e.g. regression coefficient) AND variation (e.g. standard deviation) or associated estimates of uncertainty (e.g. confidence intervals)
☐	☒	For null hypothesis testing, the test statistic (e.g. *F*, *t*, *r*) with confidence intervals, effect sizes, degrees of freedom and *P* value noted<br>*Give P values as exact values whenever suitable.*
☒	☐	For Bayesian analysis, information on the choice of priors and Markov chain Monte Carlo settings
☒	☐	For hierarchical and complex designs, identification of the appropriate level for tests and full reporting of outcomes
☒	☐	Estimates of effect sizes (e.g. Cohen's *d*, Pearson's *r*), indicating how they were calculated

Our web collection on statistics for biologists contains articles on many of the points above.

Software and code

Policy information about availability of computer code

Data collection	Zeiss LSM 800 confocal microscope was used to collect the confocal images;<br>A stereoscope (Olympus MVX10) with a digital microscope camera (Olympus Dp47) was used to collect the ovary images;<br>An Olympus Dp47 camera mounted directly above the arena was connected to a computer to record the wasp's search track.
Data analysis	Image analysis: ImageJ_v1.8.0;<br>Locomotion trajectories and speed analysis: cellSens Dimension software (ver. 2. 2; Olympus);<br>Statistical analyses were performed in GraphPad Prism version 8.0 (GraphPad Software) and SPSS Statistics 26 (IBM).

For manuscripts utilizing custom algorithms or software that are central to the research but not yet described in published literature, software must be made available to editors and reviewers. We strongly encourage code deposition in a community repository (e.g. GitHub). See the Nature Portfolio guidelines for submitting code & software for further information.

Data

Policy information about availability of data

All manuscripts must include a data availability statement. This statement should provide the following information, where applicable:

- Accession codes, unique identifiers, or web links for publicly available datasets
- A description of any restrictions on data availability
- For clinical datasets or third party data, please ensure that the statement adheres to our policy

The authors declare that all data supporting the findings of this study are available within the paper and its supplementary information files. Source data are provided with this paper. Data availability statement has been included in the manuscript.

Human research participants

Policy information about studies involving human research participants and Sex and Gender in Research.

Reporting on sex and gender	Our study does not involve human research participants
Population characteristics	Our study does not involve human research participants
Recruitment	Our study does not involve human research participants
Ethics oversight	Our study does not involve human research participants

Note that full information on the approval of the study protocol must also be provided in the manuscript.

Field-specific reporting

Please select the one below that is the best fit for your research. If you are not sure, read the appropriate sections before making your selection.

[x] Life sciences [ ] Behavioural & social sciences [ ] Ecological, evolutionary & environmental sciences

For a reference copy of the document with all sections, see nature.com/documents/nr-reporting-summary-flat.pdf

Life sciences study design

All studies must disclose on these points even when the disclosure is negative.

Sample size	Sample size were predetermined based on previous related studies in this field.<br>For egg laying behavioral experiments, at least 80 animals were used in this study, which is similar to the literatures like Sadanandappa et al. PLoS Genetics, 17(3), e1009456 (2021) and Kacsoh et al. Elife 4 (2015).<br>For staining or confocal experiments, at least 20 animals were used in this study, which is similar to Chen et al. Nat. Commun. 12, 5489 (2021).<br>For mature follicle trimming analysis and Mmp2 activity measurement, at least 30 animals were used in this study, which is similar to the literatures like Deady et al., PLoS Genet. 11, e1005604 (2015) and Knapp et al., Proc. Natl. Acad. Sci. U.S.A. 114, 699-704 (2017).<br>For parasitic efficiency assay and quantitative real-time PCR, at least 3 biologically independent experiments, more than 30 animals were used, which is similar to Chen et al. Nat. Commun. 12, 5489 (2021).<br>All sample sizes are provided in each figure legend and source data. These numbers of samples were sufficient to perform a confident data analysis.
Data exclusions	No data was excluded from our analyses.
Replication	All data presented are representative of at least three independent experiments as indicated in the figure legends, and replications were successful.
Randomization	In all experiments, wasps or flies were randomly assigned to experimental groups. Specifically, The fly and wasp adults for oviposition assay, host search performance analysis, parasitic efficiency assay, ovary mature egg and size comparison, follicle rupture and Mmp2 activity assay, oviduct muscle sarcomere length measurement were randomly selected. The samples for antibody staining, qRT-PCR, dsRNA and OA injection were also randomly selected.
Blinding	Investigators were blinded to group allocation of wasps with different age or treatments for oviposition assay (short time), host search performance analysis and parasitic efficiency assay. Blinding was not required for other experiments, because sample preparation, data collection and image analysis were performed using the same conditions for all the samples regardless of their identity.

Reporting for specific materials, systems and methods

We require information from authors about some types of materials, experimental systems and methods used in many studies. Here, indicate whether each material, system or method listed is relevant to your study. If you are not sure if a list item applies to your research, read the appropriate section before selecting a response.

Materials & experimental systems

n/a	Involved in the study
☐	☒ Antibodies
☒	☐ Eukaryotic cell lines
☒	☐ Palaeontology and archaeology
☐	☒ Animals and other organisms
☒	☐ Clinical data
☒	☐ Dual use research of concern

Methods

n/a	Involved in the study
☒	☐ ChIP-seq
☒	☐ Flow cytometry
☒	☐ MRI-based neuroimaging

Antibodies

Antibodies used	anti-OA (abcam, Cat#ab37092, rabbit polyclonal, 1:500);<br>anti-NPF (gift from Dr. Zhangwu Zhao, rabbit polyclonal, 1:500);<br>Alexa Fluor 594 Goat anti-Rabbit second antibody (Invitrogen, Cat#A11012, 1:1000);<br>ProLong Gold Antifade Mountant with DAPI (Invitrogen, Cat# P36941).
Validation	Rabbit anti-OA: reactivity validated by abcam, validated in immunohistochemistry (under product specification; 1:1,000-1:20,000; https://www.abcam.com/ab37092.pdf);<br>Rabbit anti-NPF: reactivity validated by Dr. Zhangwu Zhao, China Agricultural University (Validated in He et al. PLoS One, 8(9): e74237 (2013);<br>Alexa Fluor 594 Goat anti-Rabbit second antibody: reactivity validated by Invitrogen; validated in immunocytochemistry (ThermoFisher Sci website, under product specification);<br>ProLong Gold Antifade Mountant with DAPI: reactivity validated by Invitrogen, validated in immunocytochemistry (ThermoFisher Sci website, under product specification).

Animals and other research organisms

Policy information about studies involving animals; ARRIVE guidelines recommended for reporting animal research, and Sex and Gender in Research

Laboratory animals	All Drosophila and wasp strain information is provided in the Methods under the section "Insects".<br>Drosophila strain: Drosophila suzukii (local strain, Shandong Province, China); D. melanogaster (canton-s) and other genotypes (all the genotypes were listed in the the section "Insects"). 3-day-old Drosophila adults were used in this study.<br>Wasp strain: Leptopilina boulardi (G486), L. heterotoma (Lh14), Asobara japonica (local strain, Zhejiang Province, China), Chouioia cunea (local strain, Henan Province, China) and Scleroderma guani (local strain, Henan Province, China). 2-day-old wasp male and 2/4/6/8/10/12-day-old wasp female adults were used in this study.
Wild animals	The study did not involve wild animals.
Reporting on sex	Both male and female insects were used in this study.
Field-collected samples	The study did not involve samples collected from field.
Ethics oversight	No ethical approval.

Note that full information on the approval of the study protocol must also be provided in the manuscript.
